# Encapsulated L-Lysine: from physicochemical properties to broiler responses

**DOI:** 10.1016/j.vas.2026.100789

**Published:** 2026-07-27

**Authors:** Hosna Hajati, Amirhossein Alizadeh Ghamsari, Seyyed Abdollah Hosseini

**Affiliations:** Department of Animal and Poultry Nutrition and Physiology, Animal Science Research Institute of Iran, Agricultural Research, Education and Extension Organization (AREEO), Karaj, Iran

**Keywords:** Encapsulation, l-lysine, Broiler performance, Physicochemical characterization, Precision feeding

## Abstract

Precision nutrition in broiler production requires optimizing amino acid bioavailability while minimizing nitrogen excretion. Therefore, this study was conducted to evaluate the effects of dietary micro-encapsulated L-lysine on growth performance, feed efficiency, carcass traits, and selected blood metabolites in broiler chickens. A total of 600 one-day-old Arian broiler chicks were used in a completely randomized design with six dietary treatments, five replicates per treatment, and 20 birds per replicate. The treatments consisted of a control diet formulated to meet the recommended lysine requirement using conventional L-lysine HCl, and five diets in which micro-encapsulated L-lysine (EL) supplied 100%, 90%, 80%, 70%, and 60% of that in the control diet. The physicochemical properties of EL were characterized using Fourier Transform Infrared Spectroscopy (FTIR), Differential Scanning Calorimetry (DSC), and Field Emission Scanning Electron Microscopy (FESEM). FTIR and DSC analyses confirmed successful core–shell integration, showing a reduction in melting enthalpy from 107.9 to 83.37 J/g, indicating a transition toward a more stable matrix-type structure. Morphological analysis revealed a heterogeneous porous architecture with surface microcracks. Biological results showed that EL had no significant effect on final body weight or feed intake (*P* > 0.05). However, birds fed diets containing 80–100% EL exhibited a significantly lower feed conversion ratio (FCR) during the starter period (1–28 d) (*P* < 0.05). Additionally, EL at 70–80% levels increased HDL-cholesterol and reduced serum globulin concentrations compared with the control (*P* < 0.05). These findings indicate that micro-encapsulated L-lysine may improve feed efficiency during early growth and can partially replace crystalline lysine in broiler diets without negatively affecting carcass characteristics.

## Introduction

Efficient growth and nutrient utilization in broiler chickens are heavily dependent on diets balanced in protein and sufficient in digestible amino acids. Lysine, as a critical essential amino acid and typically the second-limiting amino acid in conventional corn–soybean meal–based diets, plays a pivotal role in muscle protein synthesis, feed efficiency, and overall productive performance (Contreras López et al., 2024; Sun et al., 2020). Inadequate lysine supply not only impairs growth and reduces carcass yield but also exacerbates nitrogen excretion, thereby negatively impacting both the economic profitability of poultry operations and environmental sustainability (Ghasemi et al., 2026).

To meet the nutritional requirements of modern high-yielding broilers, synthetic crystalline amino acids are routinely added to feed. However, this traditional supplementation method presents distinct physiological limitations. Because free crystalline amino acids are absorbed rapidly in the upper digestive tract, their uptake often exceeds the capacity for immediate protein synthesis. This imbalance leads to increased amino acid catabolism, reduced utilization efficiency, and higher nitrogen waste excretion (Hamid et al., 2025; Sun et al., 2020). Consequently, there is a pressing need for nutritional strategies that modulate amino acid delivery and absorption kinetics to ensure more efficient nutrient utilization.

Microencapsulation has emerged as a promising strategy to overcome the challenges associated with rapid absorption. By creating a physical and chemical barrier, microencapsulation protects amino acids from degradation in the acidic gastric environment and facilitates a controlled, delayed release in the small intestine. This mechanism aligns nutrient availability with the rate of protein synthesis, thereby enhancing metabolic utilization (Contreras López et al., 2024). Furthermore, encapsulated amino acids demonstrate improved stability against heat, moisture, and oxidation during feed processing and storage (Perera & Ravindran, 2025). Recent research indicates that this technology can stimulate nutrient transporter expression, improve gut health and intestinal morphology, and potentially reduce the requirement for synthetic amino acids by up to 20% without compromising performance (Ghasemi et al., 2026; Sun et al., 2020). Additionally, by influencing blood metabolites and nitrogen retention efficiency, encapsulation offers a mechanism to refine the metabolic utilization of dietary protein Hamid et al. (2025).

Despite these promising findings, studies specifically investigating encapsulated lysine in commercial broiler diets remain limited. While the benefits of amino acid encapsulation are well-documented, there is a distinct lack of comprehensive data linking advanced physicochemical characterization—specifically FTIR, DSC, and FESEM analysis—to actual biological outcomes. Our study addresses this gap by providing an integrated evaluation of the lysine-lipid matrix. We aimed to determine how the structural stability of the encapsulate translates into measurable growth performance, carcass characteristics, and physiological responses under practical production conditions. Therefore, the present study was designed to evaluate the effects of dietary encapsulated l-lysine on broilers, providing updated insights into this strategy as a tool to improve protein efficiency, reduce environmental impact, and enhance profitability in commercial broiler operations.

## Materials and methods

### Ethical statement

All animal care and experimental procedures were conducted in accordance with the regulations of the Iranian Ministry of Agriculture (experimental approval No ASRI-2016–95,014) and the guidelines of the Federation of Animal Science Societies (FASS, 2010). Ethical approval was obtained from the relevant institutional committee prior to the start of the experiment. The study protocol also complied with the ethical standards required by the journal and adhered to the European Union Directive on the protection of animals used for scientific purposes, as well as the recommendations of the Guide for the Care and Use of Agricultural Animals in Research and Teaching (FASS, 2010).

### Animal husbandry

A total of 600 one-day-old Arian broiler chicks were used in a completely randomized design with six treatments, five replicates per treatment, and 20 chicks per replicate. Experimental treatments included a control diet that was formulated to meet 100% of the recommended lysine requirement using conventional l-lysine HCl. Experimental diets containing graded levels of encapsulated l-lysine HCl were formulated to achieve total dietary lysine levels equivalent to 100%, 90%, 80%, 70%, and 60% of that in the control diet. Crude protein in the diets was determined according to standard AOAC International (2023) (Kjeldahl method). Total lysine was determined by HPLC following acid hydrolysis according to ISO 13,903:2005 with minor modifications. Briefly, diet samples were hydrolyzed with 6 M HCl at 110 °C for 24 h, filtered, derivatized with *o*-phthalaldehyde (OPA), and analyzed using reverse-phase HPLC with fluorescence detection. Quantification was performed using an external calibration curve prepared from analytical-grade l-lysine standards. All samples were analyzed in duplicate, and the mean value was used for reporting. The diets were formulated to remain isocaloric and isonitrogenous across treatments. Birds had ad libitum access to feed and water throughout the trial. Diets were formulated based on corn and soybean meal to meet the nutrient requirements for the Arian broiler strain. The basal diet composition and chemical analysis are presented in Tables 1, 2. The rearing house temperature started at 34 °C on day 1 and was reduced by 3 °C per week. A lighting schedule of 23L: 1D was maintained from day 1 to day 42.

**Table 1:** Feed ingredients and chemical composition of starter diets (days 1–21).

Ingredients of diets (g/kg)						
	Treatments^1^					
	Control	100 EL	90 EL	80 EL	70 EL	60 EL
Corn	53.975	53.175	53.115	53.055	52.995	52.935
Soybean meal	36.5	38.3	38.36	38.42	38.48	38.54
Corn Gluten	2.00	2.00	2.00	2.00	2.00	2.00
Oil	1.30	1.30	1.30	1.30	1.30	1.30
Sodium bicarbonate	0.20	0.20	0.20	0.20	0.20	0.20
Oyster shell	1.30	1.30	1.30	1.30	1.30	1.30
DL-Methionine	0.27	0.27	0.27	0.27	0.27	0.27
L-Lysine-HCL	0.18	0	0	0	0	0
Encapsulated l-Lysine-HCL	0	0.3	0.27	0.24	0.21	0.18
Di calcium phosphate	1.50	1.50	1.50	1.50	1.50	1.50
Salt	0.27	0.27	0.27	0.27	0.27	0.27
Vitamin and mineral Premix*	0.50	0.50	0.50	0.50	0.50	0.50
Phytase 10,000 FTU	0.005	0.005	0.005	0.005	0.005	0.005
Bentonite	1.00	0.88	0.91	0.94	0.97	1.00
**Chemical compositions of diets**						
Metabolizable energy (Kcal/kg)	2902	2908	2907	2905	2904	2902
Crude Protein (%)	22.1	22.1	22.1	22.1	22.1	22.1
Calcium (%)	0.90	0.90	0.90	0.90	0.90	0.90
Available phosphorous (%)	0.51	0.51	0.51	0.51	0.51	0.51
Digestible lysine (%)	1.30	1.30	1.17	1.04	0.91	0.78
Digestible methionine (%)	0.59	0.59	0.59	0.59	0.59	0.59
Digestible threonine (%)	0.86	0.86	0.86	0.86	0.86	0.86
Sodium (%)	0.18	0.18	0.18	0.18	0.18	0.18
Dietary electerolyte balance (meq/Kg)	246	246	246	246	246	246

⁎ provides the following per kg of diet: vitamin A 9000 IU, vitamin B_1_ 1.8 mg, vitamin B_2_ 6.6 mg, vitamin B_6_ 3 mg, vitamin B_12_ 0.015 mg, calcium pantotenate, 10 mg, folic acid 1 mg, biotin 0.1 mg, vitamin D_3_ 2000 IU, vitamin E 18 IU, vitamin K 2 mg, choline chloride 500 mg, Mn (manganese oxide) 100 mg, Zn (zinc oxide) 100 mg, Fe (ferrous sulphate) 50 mg, Cu (copper sulphate) 10 mg, Se (sodium selenite) 0.2 mg, I (calcium iodate) 1 mg.

1 Control: recommended lysine requirement using conventional l-lysine-HCl; 100EL: encapsulated l-lysine equivalent to 100% of that in the control; 90EL: encapsulated l-lysine equivalent to 90% of that in the control; 80EL: encapsulated l-lysine equivalent to 80% of that in the control; 70EL: encapsulated l-lysine equivalent to 70% of that in the control; 60EL: encapsulated l-lysine equivalent to 60% of that in the control.

**Table 2:** Feed ingredients and chemical composition of grower diets (days 22–42).

Ingredients of diets (g/kg)						
	Treatments^1^					
	Control	100 EL	90 EL	80 EL	70 EL	60 EL
Corn	62.615	62.615	62.564	62.495	62.430	62.400
Soybean meal	31.00	31.00	31.06	31.12	31.18	31.22
Corn Gluten	0	0	0	0	0	0
Soybean Oil	2.00	2.00	2.00	2.00	2.00	2.00
Sodium bicarbonate	0.2	0.2	0.2	0.2	0.2	0.2
Oyster shell	1.1	1.1	1.1	1.1	1.1	1.1
DL-Methionine	0.20	0.20	0.20	0.20	0.20	0.20
L-Lysine-HCL	0.13	0	0	0	0	0
Encapsulated l-Lysine-HCL	0	0.217	0.195	0.173	0.152	0.130
Di calcium phosphate	1.00	1.00	1.00	1.00	1.00	1.00
Salt	0.25	0.25	0.25	0.25	0.25	0.25
Vitamin and mineral Premix*	0.5	0.5	0.5	0.5	0.5	0.5
Phytase 10,000 FTU	0.005	0.005	0.005	0.005	0.005	0.005
Bentonite	1.00	0.913	0.935	0.957	0.987	1.00
**Chemical compositions of diets**						
Metabolizable energy (Kcal/kg)	3017	3021	3020	3019	3018	3017
Crude Protein (%)	18.9	18.9	18.9	18.9	18.9	18.9
Calcium (%)	0.85	0.85	0.85	0.85	0.85	0.85
Available phosphorous (%)	0.425	0.425	0.425	0.425	0.425	0.425
Digestible lysine (%)	1.10	1.10	0.99	0.88	0.77	0.66
Digestible methionine (%)	0.48	0.48	0.48	0.48	0.48	0.48
Digestible threonine (%)	0.65	0.65	0.65	0.65	0.65	0.65
Sodium (%)	0.17	0.17	0.17	0.17	0.17	0.17
Dietary electerolyte balance (meq/Kg)	223	223	223	223	223	223

⁎ provides the following per kg of diet: vitamin A 9000 IU, vitamin B_1_ 1.8 mg, vitamin B_2_ 6.6 mg, vitamin B_6_ 3 mg, vitamin B_12_ 0.015 mg, calcium pantotenate, 10 mg, folic acid 1 mg, biotin 0.1 mg, vitamin D_3_ 2000 IU, vitamin E 18 IU, vitamin K 2 mg, choline chloride 500 mg, Mn (manganese oxide) 100 mg, Zn (zinc oxide) 100 mg, Fe (ferrous sulphate) 50 mg, Cu (copper sulphate) 10 mg, Se (sodium selenite) 0.2 mg, I (calcium iodate) 1 mg.

1 Control: recommended lysine requirement using conventional l-lysine-HCl; 100EL: encapsulated l-lysine equivalent to 100% of that in the control; 90EL: encapsulated l-lysine equivalent to 90% of that in the control; 80EL: encapsulated l-lysine equivalent to 80% of that in the control; 70EL: encapsulated l-lysine equivalent to 70% of that in the control; 60EL: encapsulated l-lysine equivalent to 60% of that in the control.

### Dietary l-lysine prepration

The crystalline l-lysine-HCl used in the present study was supplied by Evonik Company (Germany). The product contained approximately 78.8% available l-lysine on an as-is basis. The l-lysine content was verified by high-performance liquid chromatography (HPLC) according to the AOAC Official Methods (2023), with minor modifications. Briefly, samples were dissolved in 0.1 M hydrochloric acid (HCl), filtered through a 0.22-µm membrane filter, and derivatized with o-phthalaldehyde (OPA) prior to analysis. Chromatographic separation was carried out on a reverse-phase C18 column (250 × 4.6 mm, 5 μm particle size) using gradient elution with sodium acetate buffer and an acetonitrile–methanol–water mobile phase at a flow rate of 1.0 mL min⁻¹. OPA derivatives were detected by fluorescence (excitation 340 nm; emission 455 nm), and l-lysine concentration was determined using an external calibration curve prepared with analytical-grade l-lysine standards.

To enhance the thermal stability and gastrointestinal delivery of lysine, the crystalline l-lysine-HCl was subsequently microencapsulated by Soroush Sabzeh Alborz Co. (Karaj, Iran). The encapsulation process consisted of a sequential coating system in which lysine particles were first coated with a carbohydrate-based layer followed by an outer lipid layer. According to the manufacturer's technical specifications, this coating system was designed to maintain product stability during feed processing at temperatures of up to approximately 80 °C while providing protection against premature release. The detailed formulation and processing conditions were proprietary to the manufacturer and were therefore not available for disclosure. Because of the addition of the carbohydrate and lipid wall materials, the final microencapsulated product contained approximately 60% available l-lysine on an as-is basis.

The l-lysine concentration of the microencapsulated product was determined by HPLC following acid extraction. Approximately 250 mg of the sample was mixed with 25 mL of 0.1 M HCl, vortexed, and sonicated in an ultrasonic bath for 30 min at 40 °C. After cooling at 4 °C for 20 min, the suspension was centrifuged at 10,000 × g for 10 min. The supernatant was collected, and the remaining residue was re-extracted under identical conditions. The combined extracts were adjusted to a final volume of 50 mL with 0.1 M HCl, sequentially filtered through 0.45- and 0.22-µm membrane filters, derivatized with OPA, and analyzed by reverse-phase HPLC with fluorescence detection. Quantification was performed using an external calibration curve prepared with analytical-grade l-lysine standards. The analytical procedure, including extraction efficiency, was verified by spike–recovery testing according to AOAC Official Methods (2023).

### Growth performance

Body weight and feed intake were recorded weekly for all birds in each experimental unit. Body weight (BW) and feed conversion ratio (FCR) were calculated according to Alqhtani et al. (2022). At the end of the experiment, the productive efficiency index (PEI) was calculated following Alkhulaifi et al. (2022). Alizadeh-Ghamsari et al. (2025);

### Carcass characteristics

On day 42, after a 6-hour fasting period, four birds per replicate were selected and euthanized by cervical dislocation. Carcasses were defeathered and eviscerated. Weights of the carcass (excluding skin, feathers, feet, and head), thigh + drumstick, breast, back, wings, and abdominal fat were recorded and expressed as a percentage of live body weight according to Alqhtani et al. (2022); Soares et al. (2017).

### Humoral immunity

Humoral immune response was assessed by measuring antibody titers against Newcastle disease and influenza on day 42. Blood samples from four birds per replicate were collected from the wing vein into non-heparinized tubes, centrifuged at 3000 × g for 10 min, and serum was separated into Eppendorf tubes. Hemagglutination inhibition (HI) tests were performed according to Tavakoli et al. (2021).

### Blood metabolites

On day 42, serum samples from three birds per replicate were analyzed for triglycerides, total cholesterol, LDL, HDL, and liver enzymes (AST, ALT, ALP) using commercial kits (Roche Diagnostics, Switzerland) with an autoanalyzer (HITACHI 902) according to Safaa (2013). Blood samples from four birds per replicate were collected in heparinized tubes for hematology, including RBC, hemoglobin (Hb), platelets (Pl), WBC, and hematocrit (HCT), following Kim et al. (2013). Blood smears were stained with Giemsa for differential WBC counts as described by Koochakzadeh et al. (2018).

### Differential scanning calorimetry (DSC)

Thermal properties of conventional and microencapsulated lysine were determined using a DSC 214 Polyma (NETZSCH, Selb, Germany). Approximately 5–8 mg of each sample was accurately weighed into sealed aluminum pans and heated from 10 to 300 °C at a rate of 10 °C/min under a nitrogen atmosphere (40 mL/min). Temperature and enthalpy calibrations were performed using indium standards according to the manufacturer's recommendations. Thermograms were analyzed using Proteus® software (NETZSCH, Germany) to determine thermal transitions and assess the thermal stability of the samples (ASTM International, 2018).

### Fourier transform infrared spectroscopy (FTIR)

FTIR spectra of conventional and encapsulated lysine were recorded in transmission mode using a Thermo Nicolet Avatar FTIR 380 spectrophotometer (Madison, Wisconsin, USA) over 400–4000 cm^-1^. Approximately 10 mg of sample was mixed with 90 mg KBr, ground, and pressed into pellets. Spectra were analyzed using OMNIC 9.x software (Coates, 2006).

### Field emission scanning electron microscopy (FESEM)

Surface morphology of conventional and encapsulated lysine was examined using a TESCAN MIRA4 FESEM (Brno, Czech Republic) at × 5000 magnification and 20 kV accelerating voltage. Samples were sputter-coated with 10 nm gold before imaging (Goldstein et al., 2018).

### Statistical analysis

One-way ANOVA (proc GLM) was used to test the effects of encapsulated lysine on different traits by considering a completely randomized design (CRD) (SAS Institute Inc., 2004). The mean of each replicate means was applied as experimental unit. The means were compared by Duncan's multiple range test (Duncan, 1955). To determine linear and quadratic relationships among different levels of encapsulated l-lysine, orthogonal contrasts were performed. Statistical significances were stated based on *P <* *0.05*.

## Results

### Growth performance, european production efficiency factor

The effects of dietary encapsulated lysine on growth performance of broiler chickens are presented in Table 3. No significant differences were observed in body weight (BW) or feed intake (FI) among treatments throughout the study period (*P* > 0.05), indicating that varying levels of encapsulated lysine did not compromise overall growth. During days 1–28, birds receiving 80%–100% of the recommended lysine exhibited significantly lower feed conversion ratio (FCR) compared to those fed 60%–70% of recommended lysine (*P* < 0.05). This result indicates a potential improvement in feed efficiency during the starter phase when moderate to high levels of encapsulated lysine were provided. However, over the entire rearing period (days 1–35 and 1–42), no significant differences in FCR were observed among treatments (*P* > 0.05), suggesting that the early advantage in feed efficiency did not persist throughout the entire rearing period. Livability and productive efficiency index (PEI) were also unaffected by treatment (*P* > 0.05), suggesting that the inclusion of encapsulated lysine at various levels does not negatively affect overall flock performance or survival.

**Table 3:** Effect of the experimental diets on the growth performance of broiler chickens (n = 100).

	Treatments^1^							P-value		
	Control	100 EL	90 EL	80 EL	70 EL	60 EL	SEM^2^	Combined	Linear	Quadratic
**1–7 d**										
BW (g/bird)	162.5	164.9	167.2	161.7	165.3	162.8	5.00	0.954	0.556	0.989
FI (g/bird)	162.7	151.3	158.8	138.7	161.3	169.4	11.36	0.173	0.087	0.562
FCR (g/g)	1.003	0.924	0.952	0.861	0.982	1.049	0.079	0.393	0.111	0.639
**1–14 d**										
BW (g/bird)	375.3	377.2	373.7	377.1	378.6	373.3	13.45	0.994	0.883	0.924
FI (g/bird)	445.6	458.3	469.3	475.9	474.2	476.6	18.78	0.640	0.729	0.118
FCR (g/g)	1.19	1.21	1.25	1.26	1.25	1.28	0.041	0.388	0.792	0.073
**1–21 d**										
BW (g/bird)	799.7	775.3	786.6	801.4	798.4	779.2	49.3	0.948	0.624	0.689
FI (g/bird)	1036.9	1018	1042.8	1100.5	1103.7	1080.7	61.33	0.430	0.479	0.057
FCR (g/g)	1.302	1.312	1.324	1.373	1.381	1.386	0.042	0.076	0.527	0.011
**1–28 d**										
BW (g/bird)	1209	1196.6	1197.3	1199.3	1221.0	1180.1	77.6	0.978	0.919	0.934
FI (g/bird)	1758.6	1659.5	1661.8	1673.8	1853.6	1841.5	109.93	0.095	0.029	0.393
FCR (g/g)	1.457^ab^	1.387^b^	1.390^b^	1.395^b^	1.518^a^	1.561^a^	0.045	0.002	0.002	0.241
**1–35 d**										
BW (g/bird)	1824.0	1838.8	1826.1	1835.1	1862.2	1801.8	92.89	0.974	0.873	0.759
FI (g/bird)	2814.1	2660.1	2842.3	2818.8	2947.5	3019.8	168.03	0.387	0.171	0.191
FCR (g/g)	1.539	1.451	1.557	1.535	1.581	1.678	0.051	0.058	0.048	0.122
**1–42 d**										
BW (g/bird)	2466.1	2444.0	2438.4	2456.3	2525.5	2325.4	114.4	0.427	0.786	0.833
FI (g/bird)	4409.9	4092.5	4292.2	4419.8	4465.9	4428.6	185.4	0.350	0.070	0.167
FCR (g/g)	1.797	1.675	1.761	1.806	1.769	1.915	0.070	0.150	0.050	0.297
Livability (%)	91.43	93.26	95.43	90.95	94.38	95.02	3.26	0.54	0.673	0.656
**PEI**	302.5	317.2	286.6	271.5	322.0	277.3	27.5	0.313	0.743	0.369

^a-b^Means within rows with no common letters are significantly different at *P* < 0.05.

1 Control: recommended lysine requirement using conventional l-lysine-HCl; 100EL: encapsulated l-lysine equivalent to 100% of that in the control; 90EL: encapsulated l-lysine equivalent to 90% of that in the control; 80EL: encapsulated l-lysine equivalent to 80% of that in the control; 70EL: encapsulated l-lysine equivalent to 70% of that in the control; 60EL: encapsulated l-lysine equivalent to 60% of that in the control.

2 Standard error of means. BW: body weight; FI: feed intake; FCR: feed conversion ratio; PEI: productive efficiency index.

Linear and quadratic contrasts showed that during the early growth periods (1–7 and 1–14 d), the progressive replacement of crystalline lysine with encapsulated lysine did not result in significant linear or quadratic responses for body weight (BW), feed intake (FI), or feed conversion ratio (FCR) (*P* > 0.05). Similarly, from 1 to 21 d of age, BW and FI were not influenced by either linear or quadratic trends (*P* > 0.05). However, FCR exhibited a significant quadratic response (*P* = 0.011), suggesting a non-linear relationship between the level of encapsulated lysine substitution and feed efficiency during this period. In the 1–28 d period, increasing levels of encapsulated lysine substitution were associated with significant linear responses in FI (*P* = 0.029) and FCR (*P* = 0.002), while no quadratic effects were detected (*P* > 0.05). Body weight remained unaffected by both linear and quadratic contrasts (*P* > 0.05). For the 1–35 d period, none of the measured performance traits showed significant linear responses (*P* > 0.05). Nevertheless, FI displayed a significant quadratic trend (*P* = 0.048), whereas BW and FCR were not influenced by quadratic effects (*P* > 0.05). Over the entire experimental period (1–42 d), a marginally significant linear response was observed for FCR (*P* = 0.050). In contrast, BW, FI, livability, and production efficiency factor (PEF) were not affected by linear trends (*P* > 0.05), and no significant quadratic responses were detected for any of the evaluated parameters (*P* > 0.05).

### Humoral immunity

Antibody titers against Newcastle disease virus (NDV) and influenza virus measured on day 42 are shown in Table 4. No significant differences were detected among treatments (*P* > 0.05), indicating that varying levels of encapsulated lysine did not alter the humoral immune response of broilers. This finding aligns with previous studies suggesting that lysine supplementation primarily influences growth and protein metabolism rather than directly modulating antibody production under standard rearing conditions.

**Table 4:** Effect of the experimental diets on the humoral immunity (antibody titer aginst Influenza and Newcastle diseases) and some blood parameters of broiler chickens on d 42 (n = 100).

	Treatments^1^							P-value		
	Control	100 EL	90 EL	80 EL	70 EL	60 EL	SEM^2^	Combined	Linear	Quadratic
Humoral immunity (Log2)	4.00	6.25	4.25	4.75	4.00	4.75	1.03	0.396	0.138	0.341
Influenza titer Newcastle titer	5.25	5.50	5.00	5.50	5.50	6.00	1.71	0.901	0.731	0.822
Cholesterol (mg/dl)	110.6^b^	119.3^ab^	107.7^b^	130.3^a^	131.67^a^	109.3^b^	2.84	0.007	0.353	0.018
Triglyceride (mg/dl)	67.0	79.3	88.3	86.7	112.3	62.3	17.65	0.254	0.057	0. 408
HDL (mg/dl)	36.7^b^	47.7^b^	43.5^b^	66.2^a^	65.8^a^	47.7^b^	3.26	0.011	0.365	0.002
LDL (mg/dl)	74.0	71.7	64.2	64.2	65.8	61.7	9.82	0.772	0.920	0.213
Globulin (g/dl)	1.9^a^	1.8^a^	1.6^ab^	1.5^b^	1.3^b^	1.4^b^	0.058	0.002	0.606	0.001
Albumine (g/dl)	1.6	1.6	1.7	1.5	1.7	1.5	0.17	0.918	0.943	0.698
Total protein (g/dl)	3.5	3.4	3.2	3.0	3.0	3.0	0.24	0.272	0.883	0.028
AST (U/L)	363.7	352.7	392.0	407.3	332.0	362.7	32.99	0.221	0.553	0.515
ALT (U/L)	5.0	5.0	5.3	3.3	6.0	2.7	1.90	0.518	0.646	0.713
ALP (U/L)	2845.7	2609.3	2900.3	2914.0	3024.0	3489.7	306.72	0.126	0.090	0.141
WBC (× 1/µl)	17,350	21,250	24,300	23,775	18,125	22,025	4867	0.616	0.273	0.508
RBC (× 10^5^/ µl)	2.475	2.725	2.550	2.525	2.500	2.575	0.359	0.892	0.371	0.595
Platelet (%)	6.61	5.12	6.06	6.85	6.30	5.06	2.35	0.377	0.516	0.443
Hemoglubin (g/dl)	10.75	12.10	10.28	11.58	10.05	11.43	1.145	0.331	0.433	0.371
Hematocrit (%)	31.75	34.00	29.75	33.50	31.25	30.00	2.12	0.342	0.352	0.503
Heterophyle (%)	19.75	15.25	17.75	19.25	19.75	14.25	3.94	0.603	0.598	0.617
Lymphocyte (%)	76.50	82.75	79.75	77.25	77.00	82.50	4.13	0.368	0.303	0.488
H/L ratio	0.633	0.442	0.598	0.580	0.640	0.484	0.118	0.572	0.645	0.636

^a-b^Means within rows with no common letters are significantly different at *P* < 0.05.

1 Control: recommended lysine requirement using conventional l-lysine-HCl; 100EL: encapsulated l-lysine equivalent to 100% of that in the control; 90EL: encapsulated l-lysine equivalent to 90% of that in the control; 80EL: encapsulated l-lysine equivalent to 80% of that in the control; 70EL: encapsulated l-lysine equivalent to 70% of that in the control; 60EL: encapsulated l-lysine equivalent to 60% of that in the control.

2 Standard error of means. HDL: High-Density Lipoprotein; LDL: Low-Density Lipoprotein; AST: Aspartate Aminotransferase; ALT: Alanine Aminotransferase; ALP: Alkaline Phosphatase; WBC: White Blood Cell; RBC: Red Blood Cell; H/ L ratio: High-Density Lipoprotein to Low-Density Lipoprotein Ratio.

The linear and quadratic responses of humoral immunity and blood parameters to the experimental diets are presented in Table 4. No significant linear effects were observed for most measured variables (*P* > 0.05). Antibody titers against influenza and Newcastle disease were not influenced by the linear increase in encapsulated lysine substitution.

### Blood metabolites

Serum biochemical parameters are presented in Table 4. Levels of triglycerides, LDL, albumin, total protein, and liver enzymes (AST, ALT, ALP) did not differ significantly among treatments (*P* > 0.05), indicating that encapsulated lysine does not adversely affect liver function or lipid metabolism. Interestingly, total cholesterol and HDL were higher in birds fed 70%–80% of recommended lysine compared with the control (*P* < 0.05), which may reflect an alteration in lipid transport and metabolism due to improved amino acid availability. Serum globulin concentrations were lower in birds receiving 60%–80% of recommended lysine compared to controls (*P* < 0.05), which may indicate variations in specific serum protein fractions; however, this change did not correspond with differences in antibody titers.

Similarly, serum cholesterol, HDL, LDL, globulin, albumin, total protein, liver enzymes (AST, ALT, and ALP), and hematological indices including WBC, RBC, hemoglobin, hematocrit, heterophils, lymphocytes, and H/L ratio were not affected by the linear trend of treatments (*P* > 0.05). However, triglyceride concentration showed a tendency toward a linear response (*P* = 0.057), indicating a gradual increase with decreasing levels of encapsulated lysine substitution. In contrast, several parameters exhibited significant quadratic responses. Serum cholesterol was affected by the quadratic effect of treatments (*P* = 0.018), with higher values observed at intermediate substitution levels. High-density lipoprotein (HDL) concentration also showed a significant quadratic response (*P* = 0.002), reaching greater values at moderate levels of encapsulated lysine substitution. In addition, serum globulin was significantly influenced by the quadratic effect (*P* = 0.001), generally decreasing as the substitution level increased. Total protein also demonstrated a significant quadratic response (*P* = 0.028). In contrast, antibody titers, LDL, albumin, liver enzyme activities, and hematological parameters were not affected by the quadratic trend of treatments (*P* > 0.05).

### Carcass characteristics

Carcass yield and relative weights of major parts, including breast, thigh + drumstick, neck + back + wing, abdominal fat, heart, spleen, bursa of Fabricius, gizzard, and liver, were evaluated on day 42 (Table 5). No significant differences were observed among treatments (*P* > 0.05). These results indicate that dietary lysine, whether encapsulated or at varying inclusion levels, did not affect body composition, organ development, or fat deposition. These findings suggest that the tested levels of encapsulated lysine were sufficient to support normal tissue deposition under the conditions of this study.

**Table 5:** Effect of the experimental diets on the carcass characteristics of broiler chickens on d 42 (n = 100).

	Treatments^1^							P-value		
	Control	100 EL	90 EL	80 EL	70 EL	60 EL	SEM^2^	Combined	Linear	Quadratic
Carcass yield^1^ (%)	73^.^03	72.26	72.51	70.19	73.03	72.06	1.23	0.438	0.735	0.803
Breast yield^2^ (%)	22.84	22.08	21.46	21.25	22.90	22.11	3.06	0.794	0.407	0.201
Thigh+drumstick^2^(%)	23.40	22.96	23.95	22.99	22.53	23.69	1.88	0.524	0.946	0.273
Neck+back+wing^2^ (%)	26.70	26.87	26.77	25.82	27.59	25.99	0.511	0.194	0.672	0.241
Abdominal fat^2^ (%)	1.004	0.938	1.174	0.935	0.917	0.921	0.149	0.700	0.177	0.175
Heart^2^ (%)	0.489	0.591	0.533	0.624	0.555	0.576	0.072	0.317	0.749	0.061
Spleen^2^ (%)	0.115	0.110	0.104	0.140	0.110	0.096	0.007	0.322	0.729	0.753
Bursa of fabricius^2^ (%)	0.125	0.083	0.104	0.084	0.076	0.124	0.0167	0.410	0.135	0.810
Gizzard^2^ (%)	2.132	2.406	2.425	2.450	2.277	2.316	0.342	0.968	0.390	0.398
Liver^2^ (%)	2.037	2.077	2.155	2.303	1.995	2.223	0.108	0.160	0.742	0.876

1 Control: recommended lysine requirement using conventional l-lysine-HCl; 100EL: encapsulated l-lysine equivalent to 100% of that in the control; 90EL: encapsulated l-lysine equivalent to 90% of that in the control; 80EL: encapsulated l-lysine equivalent to 80% of that in the control; 70EL: encapsulated l-lysine equivalent to 70% of that in the control; 60EL: encapsulated l-lysine equivalent to 60% of that in the control.

2 Standard error of means.

The linear and quadratic effects of replacing crystalline lysine-HCl with encapsulated lysine on carcass characteristics of broiler chickens at 42 d are presented in Table 5. No significant linear responses were observed for carcass yield, breast yield, thigh plus drumstick yield, neck plus back plus wing percentage, abdominal fat, spleen, bursa of Fabricius, gizzard, or liver (*P* > 0.05). The relative weight of the heart also was not affected by the linear trend of encapsulated lysine substitution (*P* = 0.749). These results indicate that gradual replacement of crystalline lysine-HCl with encapsulated lysine did not produce a consistent increasing or decreasing pattern in carcass traits. Similarly, most carcass characteristics were not influenced by the quadratic effect of treatments (*P* > 0.05). Carcass yield, breast yield, thigh plus drumstick yield, neck plus back plus wing proportion, abdominal fat, spleen, bursa of Fabricius, gizzard, and liver remained unchanged across dietary treatments. However, the relative weight of the heart showed a tendency toward a quadratic response (*P* = 0.061), suggesting a slight variation at intermediate substitution levels, although the effect was not statistically significant. Overall, the substitution of crystalline lysine-HCl with encapsulated lysine did not markedly affect carcass composition or organ weights in broiler chickens at 42 d of age.

### Differential scanning calorimetry (DSC)

**Fig. 1:**
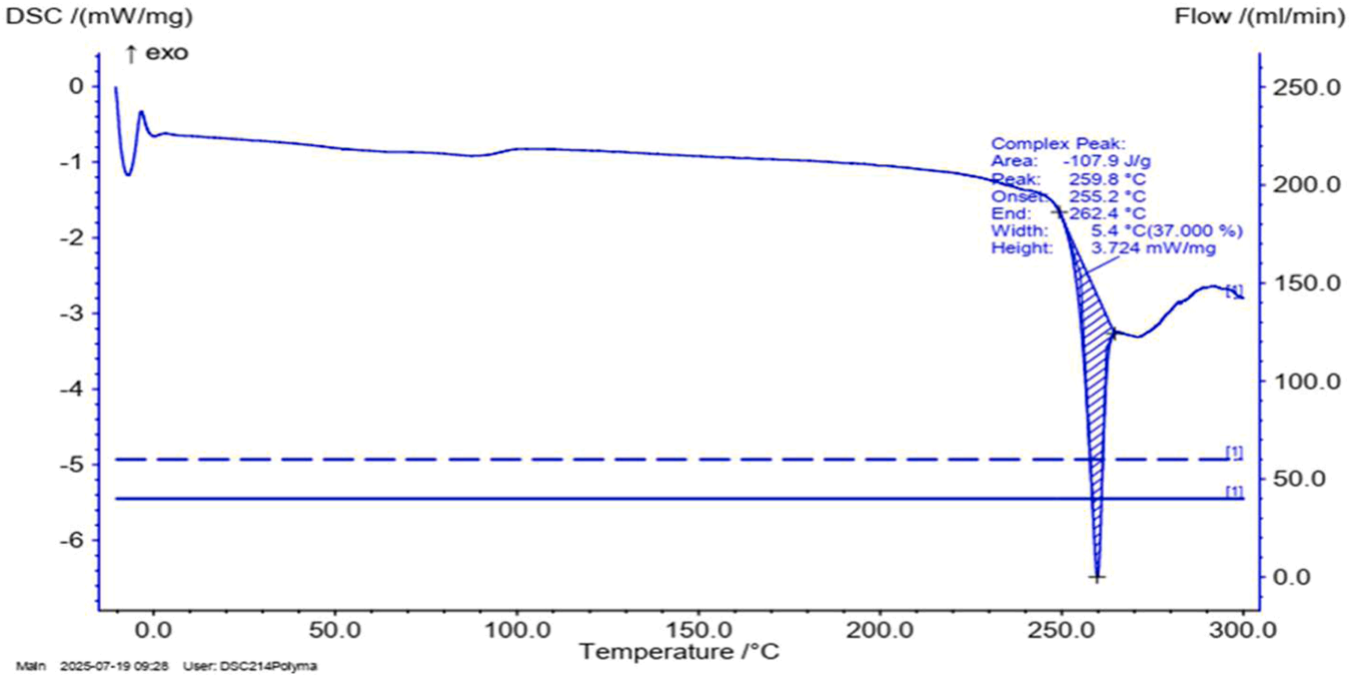
DSC thermogram of lysine.

**Fig. 2:**
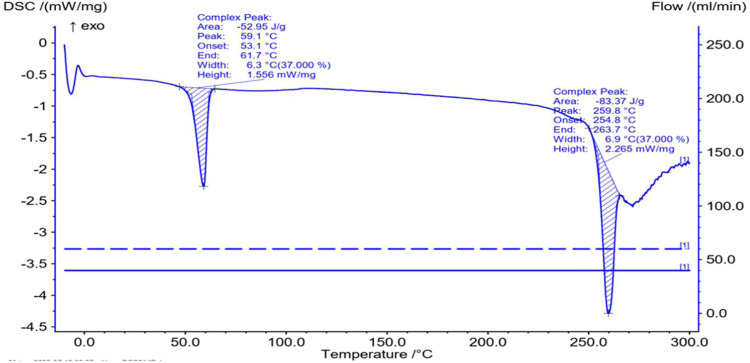
DSC thermogram of encapsulated lysine.

Thermal analysis by DSC revealed distinct melting characteristics of conventional and encapsulated lysine. Conventional lysine exhibited a sharp endothermic peak with onset at 255.2 °C, peak at 259.8 °C, and melting enthalpy of 107.9 J/g (Fig. 1). The narrow peak width (5.4 °C) indicated uniform crystallinity and high purity. Encapsulated lysine showed two endothermic events (Fig. 2): the first at 53.1–61.7 °C (peak 59.1 °C, ΔH = 52.95 J/g), likely associated with residual moisture or volatile compounds from the coating process, and the second at 254.8–263.7 °C (peak 259.8 °C, ΔH = 83.37 J/g), representing the main melting of lysine. Compared to conventional lysine, the encapsulated sample displayed slightly reduced purity and broader peak width (6.9 °C), reflecting the influence of the encapsulation matrix. These data confirm that encapsulation preserves thermal stability while introducing minor alterations due to coating materials, which is beneficial for protecting lysine during feed processing and gastrointestinal passage.

### FTIR analysis

**Fig. 3:**
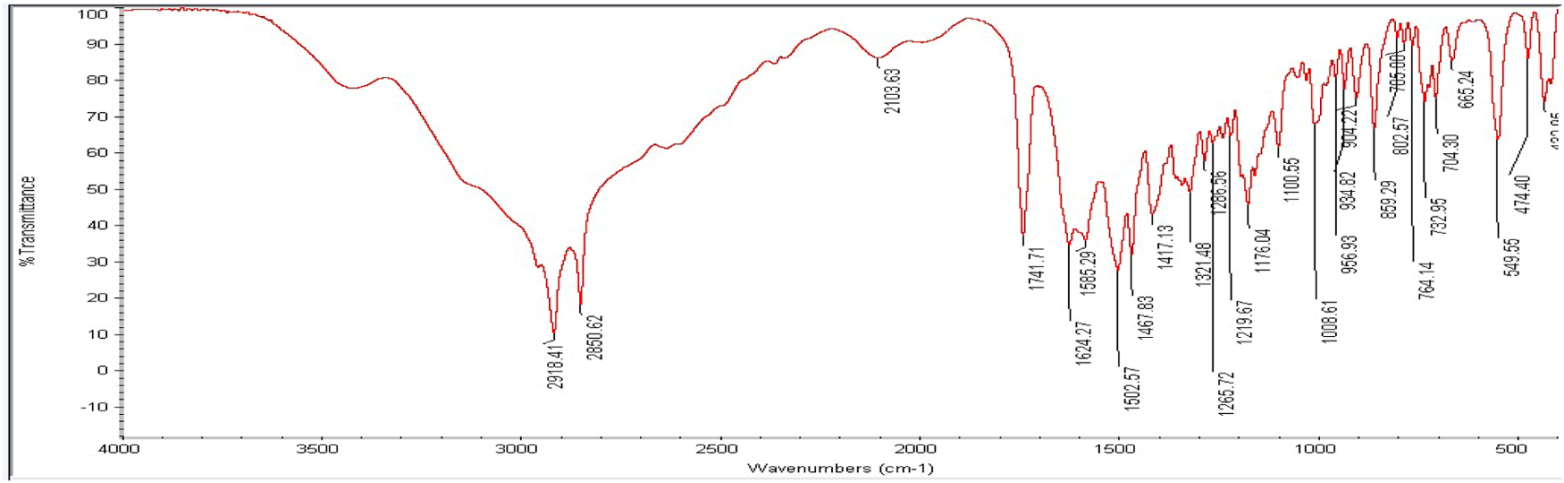
Spectrum analysis of the pure lysine. The spectrum exhibits the characteristic vibrational bands of lysine, including the broad aliphatic C–H stretching bands at 2918.41 and 2850.62 cm⁻¹, the diagnostic amino/carboxylate-related absorptions at 1624.27, 1585.29, and 1502.57 cm⁻¹, and the COO⁻ symmetric stretching band at 1417.13 cm⁻¹, confirming the structural integrity and crystalline nature of the pure amino acid.

FTIR analysis was conducted to assess the chemical stability of the lysine core and its interaction with the shell material. The spectrum of the pure lysine (Fig. 3) served as the reference, displaying its signature vibrational bands. The peak at 2942.13 cm⁻¹ is characteristic of aliphatic C–H stretching, while the sharp, intense signals at 1624.49 cm⁻¹ and 1502.28 cm⁻¹ correspond to the asymmetric and symmetric bending vibrations of the protonated amino group (NH₃⁺), respectively. These peaks confirm the zwitterionic structure and high purity of the crystalline amino acid.

**Fig. 4:**
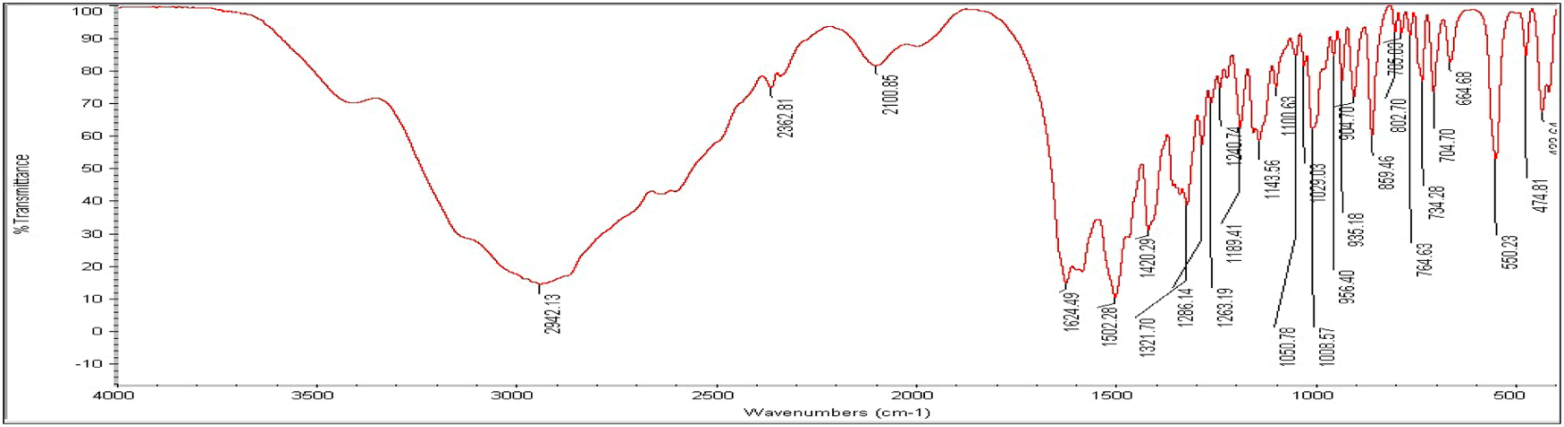
FTIR spectrum of the encapsulated lysine. The spectrum confirms the chemical integrity of the lysine core after the encapsulation process. Key vibrational signatures of the amino acid are clearly preserved, specifically the NH3^+^ asymmetric bending at 1624.49 cm⁻¹ and symmetric bending at 1502.28 cm⁻¹, along with the aliphatic C–H stretching at 2942.13 cm⁻¹. The stability of these functional groups indicates that the lysine molecules remained chemically intact and did not undergo molecular degradation during the encapsulation procedure.

Subsequently, the spectrum of the encapsulated lysine (Fig. 4) was analyzed to identify structural modifications. A prominent new absorption band at 1741.71 cm⁻¹ was observed, which is attributed to the C=O stretching of ester groups within the lipidic wall material. Additionally, the aliphatic region in Fig. 4 shows a strong doublet at 2918.41 and 2850.62 cm⁻¹, representing the CH₂ stretching vibrations of the long-chain fatty acids in the encapsulant. Despite these dominant coating signals, the primary NH₃⁺ bending vibration of lysine remained detectable at 1624.27 cm⁻¹. The preservation of this core-specific peak demonstrates that the encapsulation process successfully protected the amino acid without compromising its fundamental chemical integrity.

### SEM analysis and morphological characterization

**Fig. 5:**
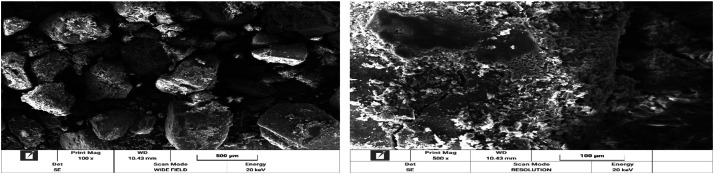
SEM micrographs of the encapsulated lysine formulation, illustrating a heterogeneous morphology, particle agglomeration, and surface micro-cracks.

**Fig. 6:**
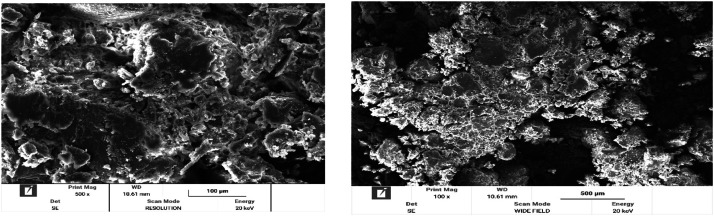
SEM micrographs of the lysine formulation, illustrating a heterogeneous morphology, particle agglomeration, and surface micro-cracks.

SEM micrographs revealed that both conventional and encapsulated lysine exhibit heterogeneous particle size distributions with irregular shapes, agglomeration, and micro-cracks (Fig. 5, Fig. 6). The particle surfaces were porous and rough, likely reflecting partial aggregation during drying or lyophilization. In encapsulated lysine, the coating matrix contributed to additional surface irregularities. Despite these morphological variations, encapsulation may influence nutrient release characteristics, which may potentially delay solubilization and modify the release pattern of lysine in the gastrointestinal tract. The observed porosity may be advantageous for modulating release kinetics, but excessive irregularity could lead to localized burst release. Overall, SEM analysis confirms the successful encapsulation of lysine, providing structural protection without altering the core amino acid.

## Discussion

### Growth performance, European production efficiency factor

The absorption of synthetic amino acids often occurs more rapidly than that of protein-bound amino acids, which can lead to temporary imbalances in plasma amino acid levels and reduced overall bioavailability (Yang & Liao, 2019). Encapsulation is a promising strategy to target nutrient delivery to specific segments of the gastrointestinal tract, allowing gradual release of amino acids at the optimal site for absorption (Gao et al., 2019). Protected amino acids are released primarily in the small intestine, resulting in a prolonged presence in the bloodstream and improved utilization efficiency between meals (Piva et al., 2007; Prandini et al., 2013). In the current study, feeding broilers encapsulated lysine at 80% of the recommended level during days 1–28 significantly improved feed conversion ratio (FCR). This indicates that early supplementation with encapsulated lysine can optimize feed efficiency without compromising growth. These results indicate that encapsulated lysine at 80% of the recommended level supported efficient feed utilization during the starter phase without compromising growth performance. In contrast, diets containing 60% or 70% of recommended lysine led to higher FCR compared with 80–100% levels, highlighting the importance of adequate lysine availability during early growth. Over the entire rearing period (days 1–35 and 1–42), however, no significant differences in growth performance or production efficiency index were observed among birds receiving 60–100% of the recommended lysine. These results suggest that even lower levels of encapsulated lysine may be sufficient to maintain overall productive performance, making it a suitable tool for precision feeding programs aiming to balance performance and cost-effectiveness. These findings are consistent with previous reports. Sun et al. (2020) demonstrated that broilers fed encapsulated lysine and methionine at 60–80% of dietary requirements performed similarly to control birds. Likewise, the use of encapsulated amino acids in laying hens allowed a reduction in supplemental lysine and methionine without negatively affecting production (Sun et al., 2020). Collins (2021) also reported that broilers receiving encapsulated amino acids exhibited higher body weights than those fed non-encapsulated forms, with numerical improvements in FCR despite slightly higher feed intake.

Beyond poultry, encapsulation has been effectively applied in ruminant nutrition. Ali et al. (2009) reported that the inclusion of encapsulated proteins and amino acids in cattle and sheep diets improved nutrient digestibility and overall animal performance. Collectively, these studies suggest that encapsulation enhances amino acid bioavailability and utilization efficiency, offering both nutritional and economic advantages in intensive livestock production systems.

### Humoral immunity

In poultry, the first line of defense against pathogens is the innate immune system, which provides immediate protection upon pathogen entry. Components of innate immunity include physical and chemical barriers, immune cells such as dendritic cells, macrophages, and natural killer cells, as well as receptors, complement proteins, and cytokines. Innate immune responses act more rapidly than adaptive immunity and are essential for the initial control of viral infections, including influenza and Newcastle disease virus. However, both innate and adaptive immunity are required to maintain overall health and effective pathogen clearance (Yang et al., 2023). The humoral immune system plays a key role in maintaining immune homeostasis and generating antibodies that neutralize pathogens. B cells are central to humoral immunity, producing antibodies in response to infectious agents (Hua & Hou, 2013). B cell-mediated immune responses can be classified as thymus-dependent or thymus-independent. Similar to T cells, activation of B cells by thymus-dependent antigens requires three signals: antigen recognition, co-stimulatory interactions with helper T cells, and cytokine-mediated signaling (Depoil et al., 2009; Kuchen et al., 2007). Physiologically, B cells present antigens or stimulatory signals to T cells, which in turn provide secondary signals to fully activate B cells (Parker, 1993). Recent studies have highlighted the potential of encapsulated nutrients in enhancing immune responses. Tripathy et al. (2021) reported that encapsulated nutrients can promote immunity by supporting cellular and humoral responses against various microorganisms. Lysine, an essential amino acid, is critical for protein synthesis, muscle deposition, cytokine production, lymphocyte proliferation, and overall immune competence. Lysine deficiency has been shown to negatively affect both antibody production and cell-mediated immunity in poultry (Geraert & Mercier, 2010). In the present study, no significant differences were observed in antibody titers against influenza or Newcastle disease on day 42 of age (*P* > 0.05). This result may be attributed to the healthy status of the birds, limited antigen exposure to systemic circulation, or the timing of sample collection. These findings suggest that under standard rearing conditions, encapsulated lysine supplementation at the tested levels did not significantly alter humoral immune responses.

### Blood metabolites

Different levels of encapsulated lysine did not significantly affect the levels of triglycerides, LDL, albumin, total protein, AST, ALT, or ALP in broiler chickens. These results are consistent with Collins (2021), who reported no significant differences in plasma total protein levels in broilers fed encapsulated amino acids. Although improved amino acid utilization would be expected to increase plasma protein levels, no such effect was observed in the current study (Collins, 2021). Similarly, Sun et al. (2020) found that blood ALT, AST, and total free amino acid levels in broilers fed diets containing encapsulated lysine did not differ from those in control birds. Interestingly, broilers receiving encapsulated lysine at 70% or 80% of the recommended lysine level exhibited higher cholesterol and HDL levels compared with the control group. The increase in HDL concentration may reflect alterations in lipid transport associated with amino acid metabolism, suggesting that moderate levels of encapsulated lysine could contribute to improved overall bird health. The increase in HDL concentration may reflect alterations in lipid transport associated with amino acid metabolism. In contrast, blood globulin levels were lower in birds fed 60–80% of the recommended lysine compared with controls. Previous studies have shown that vaccination against viral diseases such as infectious bronchitis, Newcastle disease, or infectious bursal disease can increase globulin concentrations in broilers (Kudair & Al-Hussary, 2010). Therefore, the observed changes in globulin levels may reflect a modulatory effect of encapsulated lysine on the immune system rather than a negative impact.

### Carcass characteristics

Dietary lysine plays a central role in regulating protein synthesis and muscle accretion in broiler chickens, and its impact on carcass characteristics has been widely documented. Several studies have demonstrated that increasing dietary levels of lysine, particularly in combination with methionine, can enhance breast muscle deposition and simultaneously reduce excessive fat accumulation, especially abdominal fat, in male broiler chickens (Rakangtong & Bunchasak, 2011). These responses are generally associated with improved amino acid balance, which promotes more efficient nutrient utilization and directs metabolic energy toward lean tissue growth rather than lipid deposition. In the present study, however, supplementation with encapsulated lysine at different inclusion levels did not result in statistically significant changes in overall carcass yield or in the relative weights of major carcass components, including breast meat, thigh and drumstick, neck, back and wing portions. Likewise, no noticeable effects were observed in the weights of key internal organs such as the heart, spleen, bursa of Fabricius, gizzard, and liver at 42 days of age. The absence of significant differences may suggest that the basal diet already adequately met the lysine requirement for optimal carcass development, limiting the potential for further improvement through additional supplementation or modification of lysine form. In contrast to these findings, Sun et al. (2020) reported a reduction in breast muscle yield in broilers receiving diets supplemented with encapsulated lysine and methionine at 80% and 60% of the basal requirement. Such discrepancies among studies may be attributed to several interacting factors, including differences in genetic background of the broiler strains, variations in diet formulation and amino acid balance, environmental and management conditions, as well as differences in health status and metabolic responses of the birds. These factors collectively highlight the complexity of nutrient–growth interactions and suggest that the response to encapsulated amino acids may not be uniform across all production systems.

### Thermodynamic transitions and crystallinity (DSC insights)

The DSC analysis demonstrated that microencapsulation modified the thermal properties of lysine while preserving its characteristic melting behavior. Pure lysine exhibited a sharp endothermic transition near 260 °C, consistent with the thermal profile reported for highly crystalline amino acid hydrochlorides and indicative of an ordered crystal structure with high purity (Höhne et al., 2003). The narrow melting range observed in the present study further supports the existence of a relatively homogeneous crystalline phase. Encapsulation resulted in a lower melting enthalpy and a broader melting transition compared with pure lysine. Similar reductions in enthalpy have been reported for encapsulated amino acids and other bioactive compounds, where interactions between the active core and surrounding wall materials partially disrupt crystal packing and reduce structural order (Jyothi et al., 2010). Despite this reduction in crystallinity, the melting peak temperature remained essentially unchanged, suggesting that the encapsulation process did not alter the fundamental physicochemical characteristics of lysine. An additional endothermic event was observed at lower temperatures in the encapsulated sample. Comparable thermal transitions have been reported in carbohydrate- and lipid-based encapsulation systems and are generally attributed to the loss of residual moisture or thermal relaxation of carrier materials (Fang & Bhandari, 2010). The appearance of this peak only in the encapsulated treatment supports its association with the encapsulating matrix rather than the amino acid itself. The reduction in melting enthalpy may also be associated with restricted crystal growth within the coating matrix. Similar observations have been described in microencapsulated delivery systems, where physical confinement of the active ingredient leads to lower crystallinity without compromising thermal stability (Milanović et al., 2010). Such changes are often considered advantageous because reduced crystallinity may improve matrix flexibility and contribute to more controlled release characteristics. The preservation of the principal melting transition together with the modified thermal profile suggests that the coating materials provided protection against thermal stress while maintaining the structural integrity of lysine. Comparable improvements in thermal resistance have been reported for encapsulated amino acids used in feed applications, where protection during feed manufacturing and storage is required to maintain nutrient functionality (Sun et al., 2020). Therefore, the thermal behavior observed in the present study supports the potential use of the encapsulated lysine in poultry feeds subjected to thermal processing conditions such as pelleting.

### Structural integrity and chemical shielding (FTIR comparison)

The FTIR spectra were used to evaluate whether the encapsulation process affected the chemical structure of lysine and to identify the presence of functional groups associated with the coating materials. The spectrum of pure lysine showed the characteristic absorption bands expected for crystalline lysine hydrochloride, including the NH₃⁺ bending vibrations and aliphatic C–H stretching bands. These findings are in agreement with previous reports describing the infrared spectral characteristics of lysine and other amino acids (Coates, 2006). After encapsulation, several additional absorption bands became evident, particularly in regions associated with the lipid-based coating material. The appearance of the band at approximately 1742 cm⁻¹, assigned to ester carbonyl stretching, together with the stronger CH₂ stretching vibrations in the aliphatic region, indicates the presence of the encapsulating matrix around the lysine particles. Similar spectral features have been reported in lipid-containing encapsulation systems and are generally considered evidence of successful coating formation (Gharsallaoui et al., 2007). Despite the contribution of the wall materials to the overall spectrum, the characteristic NH₃⁺ absorption band of lysine remained clearly visible after encapsulation. The absence of substantial shifts in this band suggests that the amino acid maintained its original chemical structure throughout the encapsulation process. Comparable observations have been reported for encapsulated bioactive compounds, where retention of the main functional-group vibrations indicates that the interaction between the core material and the coating is primarily physical rather than the result of chemical modification (Jyothi et al., 2010). The coexistence of lysine-specific and coating-related absorption bands suggests that the wall material formed a protective layer around the amino acid while preserving its molecular identity. This is particularly important for feed applications because the effectiveness of encapsulation depends not only on coating formation but also on maintaining the biological functionality of the encapsulated nutrient. Similar FTIR patterns have been described in studies of encapsulated feed additives, where the wall matrix acts as a barrier against environmental factors such as moisture, oxygen, and heat while leaving the active compound chemically unchanged (Fang & Bhandari, 2010). Overall, the FTIR results indicate that the encapsulation process successfully incorporated lysine into the carrier matrix without detectable structural alteration of the amino acid. The preservation of the characteristic lysine bands, together with the appearance of signals associated with the coating material, supports the conclusion that the encapsulation system provided physical protection while maintaining chemical integrity.

### Morphological comparison and potential release characteristics (SEM analysis)

SEM observations showed a clear difference between the morphology of pure lysine and that of the encapsulated product. Pure lysine appeared as compact crystalline aggregates, whereas the encapsulated particles exhibited a less regular structure with visible pores, surface roughness, and occasional fractures. Similar morphological changes have been reported in microencapsulated systems, where incorporation of the active compound into a carrier matrix modifies the original crystalline appearance and leads to the formation of irregular matrix-type particles (Gharsallaoui et al., 2007; Soppimath et al., 2001). The porous and heterogeneous surface observed in the encapsulated lysine is likely related to events occurring during particle formation and drying. Previous studies have shown that solvent removal and matrix contraction can generate pores and surface irregularities, particularly in systems containing lipid or carbohydrate wall materials (Gharsallaoui et al., 2007). The morphology observed in the present study is therefore consistent with the formation of a coating layer surrounding the lysine core rather than the presence of uncoated crystalline particles. Some particles also exhibited small cracks and discontinuities on the surface. Similar features have been described in encapsulated delivery systems and are generally associated with variations in coating thickness, drying conditions, or interactions between the core and wall materials (Jyothi et al., 2010). Although release behavior was not evaluated in the present study, these structural characteristics may influence the accessibility of water to the particle interior and consequently affect the release pattern of the encapsulated amino acid under gastrointestinal conditions. The heterogeneous morphology may also have practical implications during feed manufacture and storage. Particle shape and surface characteristics can influence handling properties, physical stability, and interaction with the surrounding environment. In this regard, the formation of a continuous coating layer around lysine may contribute to improved protection against external factors while maintaining the potential for subsequent release after ingestion. Overall, the SEM micrographs confirmed that encapsulation substantially modified the physical structure of lysine. The observed morphology supports the successful incorporation of lysine within the carrier matrix and is consistent with structures reported previously for encapsulated bioactive compounds. Further studies examining release kinetics under simulated gastrointestinal conditions would provide additional information regarding the relationship between particle morphology and lysine availability.

## Conclusions

This research highlights the novelty of using a multi-analytical approach to validate encapsulation efficacy. The integration of advanced materials characterization with animal performance data demonstrates that the lipid-coated lysine maintains its chemical integrity throughout the manufacturing process, leading to improved biological outcomes. This study used FTIR, DSC, and SEM analyses to compare pure and encapsulated l-lysine hydrochloride. The results showed that encapsulation preserved the chemical structure of lysine while modifying its thermal and morphological properties, including reduced crystallinity and a more porous surface structure. Overall, the findings indicate that the encapsulation process was effective in protecting lysine and altering its physical characteristics, which may be relevant for feed processing applications. Further studies are needed to evaluate its release behavior and in vivo performance.

## Disclosure statement

The authors confirmed that there was no conflict of interest.

## Declarations

We, the undersigned authors, declare that:

1. This manuscript is original and has not been published previously, either in whole or in part.
2. The manuscript is not under consideration for publication elsewhere.
3. All authors have made substantial contributions to the study, have read and approved the final manuscript, and agree with its submission to the journal.
4. The authors declare that there are no competing financial or personal interests that could have influenced the work reported in this manuscript.
5. All applicable institutional and national guidelines for the care and use of animals were followed, and ethical approval was obtained where required.
6. All data presented in this manuscript are accurate and honestly reported.

## CRediT authorship contribution statement

**Hosna Hajati:** Writing – review & editing, Writing – original draft, Methodology. **Amirhossein Alizadeh Ghamsari:** Resources, Investigation, Conceptualization. **Seyyed Abdollah Hosseini:** Validation, Supervision.

## Declaration of competing interest

The authors declare that they have no known competing financial interests or personal relationships that could have appeared to influence the work reported in this paper.
